# Comparison of Apolipoprotein B/A1 ratio, Framingham risk score and TC/HDL-c for predicting clinical outcomes in patients undergoing percutaneous coronary intervention

**DOI:** 10.1186/s12944-019-1144-y

**Published:** 2019-11-19

**Authors:** Min Tian, Rui Li, Zhilei Shan, Dao Wen Wang, Jiangang Jiang, Guanglin Cui

**Affiliations:** 1 Division of Cardiology, Department of Internal Medicine, Tongji Hospital, Tongji Medical College, Huazhong University of Science and Technology, Wuhan, 430030 China; 2 Hubei Province Key Laboratory of Genetics and Molecular, Mechanisms of Cardiological Disorders, Wuhan, 430030 China; 3 Department of Nutrition and Department of Epidemiology, Harvard T.H. Chan, School of Public Health, Boston, MA USA

**Keywords:** Apolipoprotein B/A1 ratio, Coronary heart disease, Percutaneous coronary intervention

## Abstract

**Background:**

Apolipoproteins (Apo) are known atherogenic factors that play important roles in many mechanisms related to coronary heart disease (CHD). However, it is unclear whether the apoB/apoA1 ratio is an equal or a better predictor than the Framingham Risk Score or TC/HDL-c for predicting clinical outcomes in patients undergoing percutaneous coronary intervention.

**Methods:**

We investigated the association between Apolipoprotein B/A1 ratio and cardiovascular risk factors as well as the severity of CHD in 2256 Han Chinese patients. The potential of Apolipoprotein B/A1 ratio, Framingham Risk Score and TC/HDL-c were assessed as a marker to predict cardiovascular adverse events in a prospective subgroup of 1639 CHD patients during a 5-year follow-up.

**Results:**

In the multivariate model, adjusted odds ratios (ORs) were significant for 3-VD vs. 1-VD (OR = 2.36; 95% CI: 1.65–3.38, for the fourth vs. first quartile; P_trend_ < 0.001). The subgroup analysis showed that patients with a higher ApoB/ApoA1 ratio had an increased risk of developing multi-branch lesions and potentially suffer more cardiovascular adverse events (anginas, myocardial infarctions, heart failures, strokes, and cardiac deaths) in the future (adjusted HR =1.92; 95% CI: 1.10–3.13, for the fourth vs. first quartile). In the ROC analysis, the AUC for ApoB/A1 ratio was larger than that of Framingham Risk Score (0.604 vs. 0.543, *p* = 0.01) and TC/HDL-c (0.604 vs. 0.525, *p* < 0.01).

**Conclusion:**

Our results suggest a significant association between ApoB/ApoA1 ratio and CHD severity and cardiovascular outcomes among patients with existing CHD and ApoB/A1 ratio demonstrated a better predictive accuracy for clinical outcomes compared with Framingham Risk Score and TC/HDL-c.

## Introduction

Apolipoproteins (Apo) are the primary structural proteins for lipoprotein particles that guide lipid transportation and interact with specific receptors to facilitate uptake and deposition of lipids into tissue, and thus play a central role in cholesterol metabolism [1]. The apolipoproteinB (ApoB) is the main component of very low-density lipoproteins (VLDL), intermediate-density lipoproteins (IDL), low-density lipoproteins (LDL) and lipoprotein (a); while ApoA1 represents the major high-density lipoproteins (HDL) Apo and is the main acceptor of cholesterol when HDL transports cholesterol from the tissues to the liver to be excreted from the body. In addition, ApoB and ApoA1 transport cholesterol from liver to cells [2]. The Apolipoprotein B/A1 ratio partially reflects the cholesterol balance between potentially atherogenic and anti-atherogenic lipoprotein particles. To date, several epidemiological studies and clinical trials have suggested that high ApoB concentrations, low ApoA1 concentrations and the ApoB/A1 ratio may be better markers for the risk of coronary vascular disease (CVD) than LDL-c, HDL-c, and LDL-c/HDL-c ratio; and ApoB/A1 ratio found to be a useful predictors of cardiovascular events [3–8]. The INTERHEART study and many other previous studies also showed that the elevated ApoB/A1 ratio was a more powerful predictor than other traditional cardiovascular risk factors (including smoking, history of hypertension and diabetes mellitus, abdominal obesity, psychosocial factors, and dietary patterns) for metabolic disorders [9–11]. In concert with this, a post hoc analysis of data from 2 randomized controlled studies (the Treating to New Targets [TNT] study and the Incremental Decrease in End Points through Aggressive Lipid-Lowering [IDEAL] study) revealed that among serum lipid parameters, ApoB/A1 ratio has the strongest relationship with major cardiovascular outcomes in patients with established coronary heart disease [7]. Recently, Sarah et al. suggested that measurements of ApoB and ApoA1 were more informative about CVD risk than measurements of low-density lipoprotein cholesterol (LDL-c) and LDL-cholesterol (HDL-c); and at given levels of ApoB and ApoA1, HDL-c should not be considered to be an independent protective factor [12]. Quantitative evidence aside, whether Apolipoprotein B/A1 ratio is a marker for severity of coronary heart disease (CHD) or is involved in complex manifestations of coronary atheroma remains unknown. Therefore, the aim of our study was to examine the association between Apolipoprotein B/A1 ratio and atherosclerotic risk factors in CHD patients. We also investigated the association between Apolipoprotein B/A1 ratio and the number of vascular lesions, using the Gensini scores and the Framingham risk score as related to severity of CHD. Furthermore, we assessed the potential of Apolipoprotein B/A1 ratio as a marker to predict cardiovascular adverse events in a prospective cohort of Chinese CHD patients during a 5-year follow-up.

### Methods

#### 2.1 Subjects

A total of 2256 Han Chinese CHD cases were enrolled from hospitalized patients at Tongji Hospital and the Institute of Hypertension (Wuhan, China) between May 2009 and October 2014 (Table 1). The selection criteria, clinical and biochemical characteristics of the study subjects were described in detail in our previous report [13]. CHD was defined as one or more of the following diagnostic criteria: (1) > 50% stenosis in at least one of the major segments of coronary arteries (the right coronary artery, left circumflex, or left anterior descending arteries) assessed by coronary angiography; (2) World Health Organization criteria for elevated cardiac enzymes (troponin T, troponin I, creatine kinase-MB, aspartate aminotransferase, and glutamic pyruvic transaminase), typical ECG change (Minnesota Code 1.1 or 1.2 in ECG), and clinical symptoms; or (3) documented history of coronary artery bypass graft or percutaneous coronary intervention. Subjects with congenital heart disease, cardiomyopathy, valvular disease, and renal or hepatic disease were excluded from the study.

**Table 1:** Basic characteristics of study participants at baseline

Variables	All Patients (*n* = 2262)	Follow-up Patients (*n* = 1639)	With MACEs (*n* = 161)
Cardiovascular risk factors			
Age	60.04 ± 10.73	60.57 ± 10.81	59.45 ± 10.65
Gender, (n)men%	1675 (74.05%)	1201 (73.28%)	113 (70.19%)^*^
BMI	24.37 ± 4.08	24.34 ± 4.30	23.76 ± 3.28
Hypertension, n (%)	1301 (57.51%)	945 (57.66%)	101 (62.73%)^#^
hyperlipidemia, n (%)	149 (6.59%)	99 (6.04%)	10 (6.21%)
Diabetes, n (%)	425 (18.79%)	317 (19.34%)	47 (29.19%)^#^
Current/ex-smoker, n (%)	1149 (50.79%)	818 (49.91%)	85 (52.79%)*
Alcohol consumption (units per week), n (%)	695 (30.73%)	503 (30.69%)	45 (27.95%)*
Previous myocardial infarction, n (%)	235 (10.39%)	152 (9.27%)	18 (11.18%)*
Heart failure, n (%)	35 (1.55%)	25 (1.52%)	5 (3.11%)*
History of cerebrovascular disease, n (%)	157 (6.94%)	117 (7.13%)	14 (8.69%)*
Biomarkers			
TC (mmol/L)	4.33 ± 1.74	4.33 ± 1.66	4.31 ± 1.56
HDL-C (mmol/L)	1.07 ± 0.55	1.06 ± 0.47	1.09 ± 0.78
LDL-C (mmol/L)	2.50 ± 0.95	2.49 ± 0.93	2.52 ± 0.92*
TG (mmol/L)	1.89 ± 1.63	1.89 ± 1.56	1.84 ± 1.32
ApoA1(g/L)	1.18 ± 0.48	1.20 ± 0.55	1.15 ± 0.32
ApoB(g/L)	0.89 ± 0.49	0.90 ± 0.53	1.02 ± 0.79*
Blood pressure (mm Hg)			
Systolic	140 ± 14.17	140 ± 15.23	141 ± 15.78
Diastolic	80 ± 13.19	80 ± 13.25	84 ± 12.86*
Coronary angiography			
1-vessel disease, n (%)	683 (30.19%)	497 (30.32)	34 (21.12%)^#^
2-vessel disease, n (%)	712 (31.47%)	515 (31.42)	42 (26.08%)^#^
3-vessel disease, n (%)	867 (38.32%)	626 (38.19)	85 (52.80%)^#^

All values are presented as mean ± SD, or number (%). *BMI* body mass index, *HDL-C* high-density lipoprotein cholesterol, *LDL-C* low-density lipoprotein cholesterol, *TC* total cholesterol, *TG* triglycerides, *ApoA1* apolipoproteinA1, *ApoB* apolipoproteinB. *p < 0.05(With MACEs Vs. Follow-up Patients); #*p* < 0.01(With MACEs Vs. Follow-up Patients)

The Gensini scoring system, which was established to define the severity of coronary stenosis [14], was also used for evaluating the coronary severity. The exact procedure was reported in our previous studies [13].

This study was approved by the Institutional ethics committees of the Tongji Hospitals, and written informed consents were obtained from all study participants.

### Measurements

Anthropometric measurements including weight and height were taken with the participants wearing light clothing and no shoes. Body mass index (BMI) was calculated as the ratio of weight (kg) to the square of the height (m^2^). Blood pressure was measured with the participant in a seated position after 5 min of quiet rest. Blood tests were taken at the first time when the patients were hospitalized, and on participants fasting for at least four hours and the blood samples were collected into tubes containing 0.1% EDTA for immediate serum lipid analyses. Using fasting venous blood samples, we measured total cholesterol, HDL-c, TG, and LDL-c, on the Rocha modular DPP system according to standard procedures at the department of clinical chemistry, Tongji Hospital. Quantitative determination of ApoB and ApoAI was done by nephelometry assays (Olympus Diagnostics, Japan).

### Framingham risk score assessment

The Framingham risk score (FRS) was calculated from the National Cholesterol Education Program (NCEP) Adult Treatment Panel (ATP) III algorithm, based on six coronary risk factors: gender, age, total cholesterol, HDL-cholesterol, systolic BP and smoking habit [15]. Among these factors, age, blood pressure, and cholesterol levels, were categorized according to their values and smoking status was classified as either “current smoker” or “non-smoker”. Finally, the corresponding points were assigned to each individual and the total score was used to reflect the individual’s CHD risk level.

### Follow-up

In the present study, the primary outcome was the composite of all-cause death, stroke, myocardial infarction (MI), post-discharge revascularization (PCI/CABG), or unstable angina (UA) [16], according to the ACC/AHA cardiovascular endpoints data standards in clinical trials. The secondary end-point was event of first cardiovascular rehospitalization defined as visits to the hospital for angina-, arrhythmias-, or heart failure-related symptoms such as dyspnea and edema. The composite endpoint of cardiovascular adverse events were defined as the first occurrence of all-cause death, cardiac death, angina, new-onset/recurrent myocardial infarction, heart failure, repeat revascularization, stent thrombosis, or stroke/transient ischemic attack [17]. The follow-up information was obtained from a subset of 1639 patient during a 5-year follow-up, who were representative for the entire study population regarding baseline characteristics, by telephone or face to face interviews after the patients were discharged and the medicine treatments were also recorded at the same time. The patients who dropped out were excluded from the survival analysis because of an incomplete dataset or wrong phone numbers they had put on the medical records. Details regarding the information of the follow-up study were described in the previous reports [18, 19].

### Statistical analysis

Statistical analyses were performed with SPSS 24.0 (SPSS Inc., Chicago, Ill) for Windows (Microsoft Corp, Redmond, Wash). The sample size in the present study was calculated by R studio (version 1.1.463) using “Hsmisc” packages. For the prospective cohort, the event rate in fourth quartile of plasma apoB/apoAI ratios the was assumed to be 10, and 50% relative reduction of MACE risk in the first quartile. To achieve a power of 80% and allow for 10% losing to follow-up, 480 patients was needed in each quartiles. The sample size was calculated based on the primary end point for the CHD patients and we got a power of 0.95 to achieve the odds ratio (OR) of 1.6 for the risk of CHD. Assuming a percentage of poor responders of 5% at baseline, at least 275 patients were required (alpha and beta of 0.05). All continuous variables are expressed as mean ± standard deviation (mean ± SD). Categorical variables were presented as number and proportion (*n*, %). We used Spearman’s rank correlations to evaluation correlations between ApoB/ApoA1 ratio and cardiovascular risk factors among the CHD patients. The association between ApoB/ApoA1 ratio and Lipoprotein (Total Cholesterol/HDL-c) ratio, modified Gensini score and Framingham risk score were compared using multivariate linear regression analysis after adjustment for age, sex, BMI, smoking, drinking, hypertension and diabetes. We divided the distributions of ApoB/ApoA1 ratio and FRS among CHD patients into quartiles. The associations between the proportion of patients with 3-, 2-, 1-vessel disease and ApoB/ApoA1 ratio were compared using logistic regression analysis with adjustment for sex, age, drinking, hypertension, diabetes, smoking, and BMI in patients to estimate adjusted odds ratios (ORs) and 95% confidence intervals (CIs). Linear trend *p*-values were derived by modeling the median value of each ApoB/ApoA1 ratio quartile as a continuous variable in adjusted models. To test the interaction between ApoB/ApoA1 ratio and subgroups in association with numbers of vessel disease, we introduced a multiplicative interaction term and ApoB/ApoA1 ratio quartiles as continuous variables and added these variables to the aforementioned multivariate model. In the subgroup analyses, the predictive values of ApoB/ApoA1 ratio on outcomes during the 5-year follow-up period were analyzed by Cox proportional hazards models. Kaplan-Meier curves and Cox proportional hazards regression models were used to evaluate the associations of ApoB/ApoA1 ratio with adverse outcomes of CHD patients. The 95% confidence intervals (CIs) were computed from regression parameters. Three Cox-regression models were run as follows: without adjustment; adjusting for age, sex, and adjusting for age and sex as well as conventional clinical risk factors such as smoking, drinking, body mass index, hypertension, and diabetes; In the regression models, the ApoB/ApoA1 ratio was analyzed as categorical variables and the distributions of the ApoB/ApoA1 ratio among CHD subjects were divided into quartiles or tertils. Diagnostic accuracy of ApoB/ApoA1 ratio, FRS, and TC/HDL-c on a continuous scale in the prediction of clinical adverse outcomes of CHD was estimated as the area under the curve (AUC) from receiver operating characteristic curves. The diagnostic accuracies were determined to evaluate the ability of the 2 parameters to correctly discriminate between individuals with and without clinical outcomes. Equality of the AUC on the receiver operating curves was tested using the algorithm suggested by Delong et al. [20]. All diagrams were performed using Prism (GraphPad). All probability values were 2-sided, and a probability value less than 0.05 was considered statistical significance.

### Results

#### 3.1 ApoB/ApoA1 ratio and cardiovascular risk factors

The mean and standard deviation of ApoB/ApoA1 ratio in our study population was 0.79 ± 0.31 (Additional file 1: Figure S1). The correlation coefficients between the ApoB/ApoA1 ratio and the clinical variables are shown in Additional file 1: Table S1. The correlation coefficient (β) value with ApoB was 0.746, and with ApoA1 was − 0.491. Among the clinical variables, HDL-c (β = − 0.4), female sex (β = − 0.101), and age (β = − 0.137) showed a significant negative correlation with the ApoB/ApoA1 ratio, whereas LDL-c (β = 0.534), body mass index (BMI) (β = 0.128), systolic blood pressure (BP) (β = 0.79), total cholesterol (β = 0.414), and triglycerides (β = 0.205) were positively correlated with the ApoB/ApoA1 ratio. Based on the correlation analysis, age, sex, BMI, systolic BP, ApoB, ApoA1, current smoking, LDL-c, HDL-c, total cholesterol, and triglycerides were selected for multiple linear regression analysis. Of all the risk factors, the most informative model showed that total cholesterol, triglyceride, HDL-c, and LDL-c were found to be the main determinants of ApoB/A1 ratio.

Scatter plots displaying correlations between ApoB/A1 ratio and total cholesterol/HDL-c are shown in Additional file 1: Figure S2A. The ApoB/A1 ratio was positively correlated with total cholesterol/HDL-c (coefficient of determination: R^2^ = 0.46, β ± SE =0.15 ± 0.001, 95%CI: 0.007–0.013, *p* = 5.28 × 10^12^) (Additional file 1: Figure S2A). A similar trend was seen between ApoB/A1 ratio and Framingham Risk Score (coefficient of determination: R^2^ = 0.008, β ± SE =0.08 ± 0.02, 95%CI: 0.008–0.086, *p* = 0.019) (Additional file 1: Figure S2B). However, the fit was poor, and the scatter was wide. The concentration of both LDL-c and TG increased across the tertiles of ApoB/A1 ratio in our study (Additional file 1: Figure S3).

### 3.2 The association of ApoB/ApoA1 ratio with CHD disease severity

We explored whether the ApoB/AI ratio was associated with vascular lesions and contributed to CHD severity as well. Table 2 presents logistic regression results for the number of vascular lesions associated with the ApoB/ApoA1 ratio categorized into quartiles according to the distribution in our population. In this multivariate model, trend tests remained significant, and adjusted ORs were significant for 2VD vs.1VD (OR = 1.71; 95% CI: 1.18, 2.49, for the fourth vs. first quartile; P_trend_ = 0.026), 3VD vs. 1VD (OR = 2.36; 95% CI: 1.65, 3.38; P_trend_ < 0.001), and 3VD vs.1VD + 2VD (OR = 1.71; 95% CI: 1.28, 2.28; P_trend_ = 0.001) (Table 2). In the subgroup analysis, no clear differences were found in associations between numbers of vascular lesions and the ApoB/A1 ratio according to strata of age, smoking, drinking, and hypertension (Additional file 1: Figure S4). However, the significant effects were lost in subgroup of females, BMI ≥ 25, and diabetes. In the joint association analysis, significant interactions were found between 2VD vs.1VD and age (*p* = 0.003), BMI (*p* = 0.01), diabetes (*p* = 0.006) as well as smoking (*p* = 0.03). Furthermore, we found a significant association between the tertiles of ApoB/A1 ratios and gradually increased Gensini scores (*p* = 0.001) (Additional file 1: Figure S5A).

**Table 2:** Adjusted odds ratios [95% confidence interval (CI)] for coronary artery disease patients with the diagnosis determined by angiography according to quartiles of apoB/A1 ratios

	Quartiles of plasma apoB/apoAI ratios				
	Q1(*n* = 567)	Q2(*n* = 568)	Q3(*n* = 565)	Q4(*n* = 562)	p-Trend*
2VD Vs.1VD (712Vs.683)					
Model 1	1	1.33 (0.96–1.83)	1.23 (0.89–1.68)	1.88 (1.34–2.65)	0.001
Model 2	1	1.27 (0.91–1.75)	1.21 (0.88–1.67)	1.88 (1.33–2.66)	0.01
Model 3	1	1.24 (0.87–1.76)	1.08 (0.77–1.53)	1.71 (1.18–2.49)	0.026
3VD Vs. 1VD (867Vs.683)					
Model 1	1	1.33 (0.97–1.82)	1.13 (0.83–1.54)	2.45 (1.78–3.39)	1.49 × 10^−5^
Model 2	1	1.29 (0.94–1.77)	1.11 (0.81–1.52)	2.39 (1.72–3.31)	5.72 × 10^− 5^
Model 3	1	1.36 (0.97–1.92)	1.13 (0.80–1.59)	2.36 (1.65–3.38)	3.82 × 10^−5^
3VD Vs.1VD + 2VD (867Vs.1395)					
Model 1	1	1.14 (0.87–1.49)	1.40 (0.79–1.36)	1.68 (1.29–2.18)	4.28 × 10^−4^
Model 2	1	1.14 (0.86–1.49)	1.03 (0.78–1.35)	1.65 (1.26–2.15)	0.01
Model 3	1	1.17 (0.88–1.58)	1.05 (0.78–1.41)	1.71 (1.28–2.28)	0.001

*p-Trend across quartiles of ApoB/A1 ratios were obtained by including the median of each quartile as a continuous variable in logistic regression models. *CI* confidence interval, Model 1, without adjustment; Model 2, adjusted for age, sex; Model 3, adjusted for the variables in Model 2 and BMI, Diabetes, Hypertension, Smoking, Drinking.1VD = 1-vessel disease; 2VD = 2-vessel disease; 3VD = 3-vessel disease

In the next analysis, the ApoB/A1 ratio was examed in complex conditions of cardiovascular and cerebrovascular disease manifestations including previous myocardial infarction, heart failure, and history of cerebrovascular disease. With an increasing number of the three cardio-cerebrovascular disease manifestations, the ApoB/A1 ratio gradually showed an increasing trend. However, this effect was not statistically significant (Additional file 1: Figure S5B).

### 3.3 ApoB/A1 ratio and cardiovascular adverse events in CHD patients

**Fig. 1:**
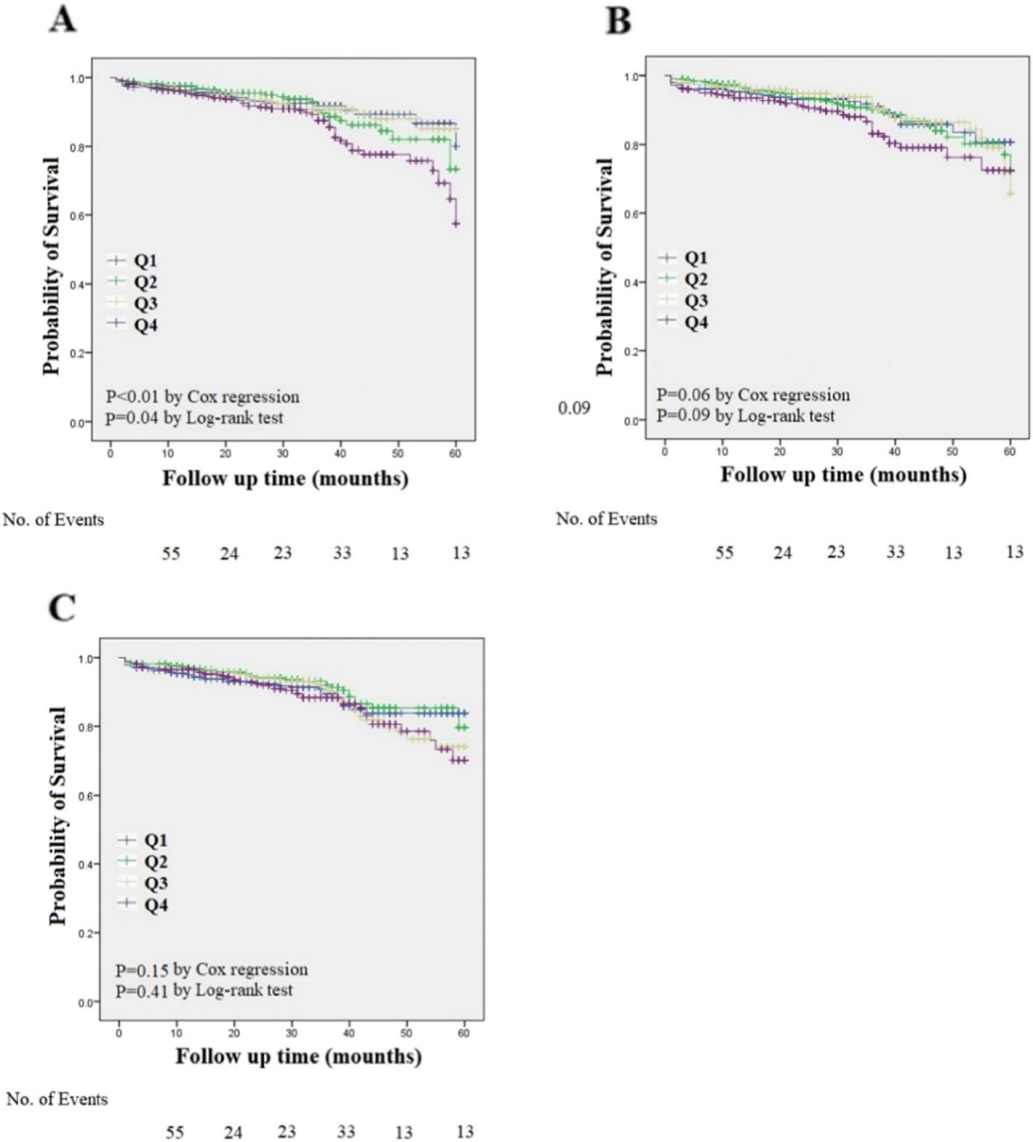
Clinical outcomes in the follow-up CHD population. Kaplan-Meier curves for the cardiovascular adverse events. X axis represents the follow-up time and the Y axis represents cumulative survival. The *P* values were calculated using the log-rank tests. **a** Kaplan-Meier curves for the cardiovascular adverse events according to the quartiles of ApoB/A1 ratios; **b** Kaplan-Meier curves for the cardiovascular adverse events according to the quartiles of FRS: Q1(*n* = 457), Q2(*n* = 721), Q3(*n* = 603), Q4(*n* = 481). **c** Kaplan-Meier curves for the cardiovascular adverse events according to the quartiles of TC/HDL-c: Q1(*n* = 566), Q2(*n* = 568), Q3(*n* = 563), Q4(*n* = 565). No. of Events: Number of clinical outcomes in the month-points

The prognostic value of ApoB/A1 ratio was evaluated in the prospective cohort of 1639 coronary heart disease patients during a period of 5-year follow-up. During the follow-up period, there were 161 adverse events, including 17 unstable anginas, 22 myocardial infarctions, 20 heart failures, 16 strokes, and 86 deaths (47 cardiac deaths) in the study population. Figure 1 shows the unadjusted associations between the ApoB/A1 ratio and cardiovascular adverse events using the Kaplan-Meier curve method, stratifying the patients according the quartiles of ApoB/A1 ratio. Increasing ApoB/A1 ratio was marginally associated with increased cardiovascular adverse events (Log rank *P* = 0.04, Fig. 1a). Among CHD patients, we also divided the distributions of the FRS and TC/HDL-c into quartiles and found no significant association between quartiles of FRS or TC/HDL-c and cardiovascular adverse events (Log rank *P* = 0.09 for FRS, Fig. 1b; Log rank *P* = 0.41 for TC/HDL-c, Fig. 1c).

Multivariable Cox regression was performed to test whether the ApoB/A1 ratio was an independent risk predictor of cardiovascular outcomes (Table 3). Increasing quartiles of the ApoB/A1 ratio were found to be a significant predictor in the Cox-regression analysis and in a multivariate model after adjustment for conventional ovariates (Q4 vs. Q1, adjusted HR =1.92; 95% CI: 1.10–3.13). The association remained significant when the ApoB/A1 ratio was treated as a continuous variable (adjusted HR for each SD increment 1.59; 95% CI: 1.29–1.96) for cardiovascular adverse events (Table 3). In the Cox-regression analysis, the Framingham Risk Score was not significantly associated with the outcome without adjustment for clinical covariates (Q4 vs. Q1, Crude HR = 1.60; 95%CI: 0.98–2.59). However, the association became significant after adjustment for conventional clinical covariates (Q4 vs. Q1, adjusted HR =2.20; 95% CI: 1.08–3.56). Similar trends were found when the Framingham Risk Score was treated as a continuous variable (Table 3). No significant associations were found between quartiles of TC/HDL-c and the outcome with or without adjustment for clinical covariates.

**Table 3:** HR and 95%CI for multivariate-adjusted Analysis of the ApoB/apoA1 ratio, TC/HDL-c or Framingham Risk Score to Long-term Clinical Outcomes

Quartiles of plasma apoB/apoAI ratios					1-SD Increasing of Variable^a^
ApoB/apoA1 ratio	Q1(*n* = 567)	Q2(*n* = 568)	Q3(*n* = 565)	Q4(*n* = 562)	
Model 1	1	1.14 (0.66–1.88)	1.12 (0.65–1.94)	1.75 (1.09–2.95)	1.55 (0.26–1.91)
Model 2	1	1.15 (0.68–1.97)	1.13 (0.65–1.97)	1.91 (1.09–2.89)	1.57 (1.28–1.93)
Model 3	1	1.25 (0.69–2.23)	1.31 (0.73–2.35)	1.92 (1.10–3.13)	1.59 (1.29–1.96)
Quartiles of TC/HDL-c					
TC/HDL-c	Q1(*n* = 566)	Q2(*n* = 568)	Q3(*n* = 563)	Q4(*n* = 565)	
Model 1	1	0.82 (0.52–1.32)	1.09 (0.70–1.69)	1.21 (0.78–1.87)	1.04 (0.89–1.23)
Model 2	1	0.78 (0.48–1.25)	1.03 (0.67–1.60)	1.17 (0.76–1.81)	1.03 (0.87–1.23)
Model 3	1	0.90 (0.52–1.56)	1.16 (0.64–2.11)	1.27 (0.56–2.89)	1.02 (0.85–1.22)
Quartiles of Framingham Risk Score					
Framingham Risk Score	Q1(*n* = 457)	Q2(*n* = 721)	Q3(*n* = 603)	Q4(*n* = 481)	
Model 1	1	1.04 (0.63–1.70)	1.00 (0.60–1.67)	1.60 (0.98–2.59)	1.04 (0.98–1.09)
Model 2	1	1.37 (0.80–2.36)	1.56 (0.84–2.56)	2.12 (1.06–3.02)	1.11 (1.04–1.18)
Model 3	1	1.34 (0.75–2.4)	1.31 (0.67–2.57)	2.20 (1.08–3.56)	1.08 (1.01–1.15)

*HR* hazard ratio, *CI* confidence interval. Model 1, without adjustment; Model 2, adjusted for age, sex; Model 3, adjusted for Model 2, BMI, Diabetes, Hypertension, Smoking, Drinking. ^a^HR indicates hazard ratio for per each SD increase of ApoB/apoA1 ratio, TC/HDL-c or Framingham Risk Score

ROC analysis for clinical outcomes at 5-year is shown in Fig. 1d. The 5-year clinical outcomes were predicted by ApoB/A1 ratio (AUC: 0.604, 95% CI: 0.550 to 0.659; *P* = 2.7 × 10^–4)^ on a continuous scale using receiver operating characteristics curves. In the ROC analysis, the AUC for ApoB/A1 ratio was larger than that of Framingham Risk Score (0.604 vs. 0.543, *p* = 0.01) and TC/HDL-c (0.604 vs. 0.525, *p* < 0.01). ApoB/A1 ratio demonstrated a better predictive accuracy for clinical outcomes compared with Framingham Risk Score and TC/HDL-c.

## 4. Discussion

The present study examined the ApoB/ApoA1 ratio, cardiovascular risk factors, and the severity of CHD in the Han Chinese population, and provided evidence of associations between the ratio and increased severity and poor prognosis of CHD. We found a significant positive correlation between ApoB/ApoA1 ratio and FRS and observed a significant association between ApoB/ApoA1 ratio and the number of vascular lesion and Gensini scores. The prospective cohort study showed that patients with higher ApoB/ApoA1 ratio had increased risk of developing multi-branch lesions and suffering more cardiovascular adverse events in the future.

In the last few decades, the association between elevated lipids levels and cardiovascular disease has been well-established. However, the extent to which components of blood lipids directly promote cardiovascular disease or represent a biomarker of risk has been debated, especially for the triglyceride and HDL-c [21, 22]. Several new markers have been introduced as alternative means to refine risk estimation beyond LDL-c from Friedewald’s formula in the presence of cardiovascular disease, such as non-HDL cholesterol, total cholesterol/HDL-c ratio, non-HDL cholesterol/HDL-c, and the apoB/apoA1 ratio [23, 24]. However, these results may have different effects in the prediction of dyslipidemia [25], cardiovascular disease [12], type 2 diabetes [26], MetS and insulin resistance [27], because the different types of lipid particles play different roles in lipid metabolism [12]. Therefore, as the ratios of cholesterol to apolipoprotein within LDL-c and within HDL-c particles vary, the positive associations of ApoB and of LDL-c with risk may differ, as may the inverse associations of apoA1 and of HDL-c with risk. On the other hand, almost all of the protein component of LDL-cholesterol is made up of ApoB, while ApoAI is important in removing excess cholesterol from tissues and incorporating it into HDL cholesterol for reverse transport to the liver, thus manifesting antiatherogenic effects [28]. The balance between proatherogenic and antiatherogenic lipoprotein particles, is a plausible way of characterizing cardiovascular risk independent of LDL-c levels. Now, routine measurement of apoB, apoA1, and their ratio ApoB/ApoA1 is widely available. In Western countries, high apoB and low apoA1 have been shown to be an independent predictor of CHD [24, 29, 30]. Sarah et al. [12]. showed that there was about seven-fold variation in the relative risk of MI between the top and bottom deciles of the ratio in UK heart disease patients. Moreover, the ApoB/apoA1 ratio was informative even in old age (70–79) and was highly reproducible, with an intraclass correlation coefficient of 0.81 between measurements on samples taken a few years apart. Furthermore, the INTERHEART case–control study [8] demonstrated the global importance of a single measurement of this ratio, and reported it to be better than any cholesterol-based ratios in predicting CHD. In the present study, we provided new evidence that ApoB/ApoA1 ratio predicts the severity and prognosis of CHD in the Han Chinese population.

Traditional cardiovascular risk factors such as smoking, obesity, low physical activity, low alcohol consumption and a diet high in sugar and low in fermented dairy products have been found to be correlated with an unfavorable Apo profile [31]. apolipoproteins B and A1 ratio has been widely used in predicting cardiovascular risk in the European [32] and American population [4]. However, few evidence reported in the Asian. Our study confirms prior results and found a significant correlation between the ratio and the FRS which was based on six coronary risk factors: gender, age, total cholesterol, HDL-cholesterol, systolic BP, and smoking habit. Indeed, almost all the parameters increased significantly in patients with 3-VD stenosis than in those in the 1VD group. However, the ApoB/ApoA1 ratio was independently associated with the number of vascular lesion and Gensini scores, which was consistent with a previous report [33]. The INTERHEART study suggested that ApoB/apoA1 ratio provided the greatest odds ratio for myocardial infarction (OR = 1.59, 95% CI 1.52–1.64, 3]. In the current study, we found a strong relationship between the ApoB/apoA1 ratio and long-term cardiovascular events among CHD patients. However, we were unable to analyze subtypes of CVD events due to small sample sizes. Otherwise, we also performed ROC analysis for clinical outcomes at 5-year. The 5-year clinical outcomes were predicted by ApoB/A1 ratio (AUC: 0.604, 95% CI: 0.550 to 0.659; *P* = 2.7 × 10^–4)^ on a continuous scale using receiver operating characteristics curves. The AUC for ApoB/A1 ratio was larger than that of Framingham Risk Score (0.604 vs. 0.543, *p* = 0.01) and TC/HDL-c (0.604 vs. 0.525, *p* < 0.01). ApoB/A1 ratio demonstrated a better predictive accuracy for clinical outcomes compared with Framingham Risk Score and TC/HDL-c (Data were not shown).

Our data must be interpreted in the context of potential limitations. Given the cross-sectional nature of some of our analyses (e.g., severity of CHD), the observed association of different lipid ratios with CHD severity may not be causal. Medication history, history of previous medical or surgical diseases, duration of diabetes, or possible in tolerance to medications were ascertained retrospectively. The condition of some patients might not be reliably assessed because of the uncertain interval from index date to the exact day of medication discontinuation, that is, the available data were only the time at when medication was discontinued at each time point of follow-up. Another limitation of the present study is we were unable to compare predictive power of ApoB/apoA1 ratio versus other ratios due to limited statistical power. Finally, it is important to confirm these findings in prospective cohort studies in other ethnic groups.

## 5. Conclusion

In conclusion, we found a significant association between ApoB/ApoA1 ratio and CHD severity and cardiovascular outcomes among patients with existing CHD. Our results strongly suggest the role of ApoB/ApoA1 ratio in the pathogenesis of CHD and these findings could potentially have significant clinical and public health implications.

## Additional files

**Additional file 1.** **Table S1.** ApoB/A1 ratio with demographic characteristics and cardiovascular risk factors within CHD patients. **Table S2.** Multiple linear regression analysis of the apoB/A1 ratios as for important covariates. **Figure S1.** Histogram distribution of ApoB/A1 ratio in our population. **Figure S2.** Association between ApoB/A1 ratio and total cholesterol/HDL and Framingham Risk Score. **Figure S3.** Associations Between Tertiles of ApoB/A1 ratio and LDL and TG levels. **Figure S4.** Adjusted odds ratios [95% confidence interval (CI)] for coronary artery disease patients with the diagnosis determined by angiography according to quartiles of ApoB/A1 ratios in subgroups. **Figure S5.** Associations between tertiles of ApoB/A1 ratio and CHD diseas severity.

## Data Availability

The original data used to support the findings of this study are available from the corresponding author upon request.

## Abbreviations

ApoA1: Apolipoprotein A1
ApoB: Apolipoprotein B
AUC: Area under the curve
BMI: Body mass index
CVD: Cardiovascular disease
HDL-C: High-density lipoprotein cholesterol
LDL-C: Lowdensity lipoprotein cholesterol
ROC: Receiver operator characteristic curve
TC: Total cholesterol
TG: Triglyceride
CHD: Coronary heart disease
ORs: Odds ratios
Cis: Confidence intervals
FRS: Framingham risk score
