# Supplementary material for: Comparison of Apolipoprotein B/A1 ratio, Framingham risk score and TC/HDL-c for predicting clinical outcomes in patients undergoing percutaneous coronary intervention
Source: Lipids Health Dis. 2019 Nov 19;18:202. doi: 10.1186/s12944-019-1144-y (PMC6864950; doi:10.1186/s12944-019-1144-y)

**Comparison of Apolipoprotein B/A1 Ratio，Framingham Risk Score and TC/HDL-c for predicting clinical outcomes in patients undergoing percutaneous coronary intervention**

Min Tian, MD^1,2^, Rui Li, MD, PhD^1,2^, Zhilei Shan，MD, PhD^3^, Dao Wen Wang, MD, PhD^1,2^, Jiangang Jiang, MD, PhD^1,2^, Guanglin Cui, MD, PhD^1,2,3^.

1. Division of Cardiology, Department of Internal Medicine, Tongji Hospital, Tongji Medical College, Huazhong University of Science and Technology, Wuhan 430030, China.

2. Hubei Province Key Laboratory of Genetics and Molecular Mechanisms of Cardiological Disorders, Wuhan 430030, China.

3. Department of Nutrition and Department of Epidemiology, Harvard T.H. Chan School of Public Health, Boston, Massachusetts, USA

Corresponding Author:

Jiangang Jiang, MD, PhD and Guanglin Cui, MD, PhD

Division of Cardiology, Department of Internal Medicine, Tongji Hospital, Tongji Medical College, Huazhong University of Science & Technology

Hubei Province Key Laboratory of Genetics and Molecular Mechanisms of Cardiological Disorders

Wuhan 430030, People’s Rep. of China

Tel. & Fax: 86-27-8366-3280

Email: jiangjg618@126.com or cuiguanglin@tjh.tjmu.edu.cn

Running title: Apolipoprotein B/A1 Ratio and coronary heart disease

Key Words: Apolipoprotein B/A1 ratio, Coronary heart disease, Severity

| Table1. ApoB/A1 ratio with demographic characteristics and cardiovascular risk factors within CHD patients. | | |
| --- | --- | --- |
| Variable | Correlation coefficien(β) | P-value |
| Age | -0.137** | 1.3×10^-9^ |
| Sex | -0.101** | 0.001 |
| Waist–hip ratio (WHR) | 0.04 | 0.094 |
| BMI | 0.128** | 1.3×10^-5^ |
| Systolic BP | 0.79** | 4.5×10^-4^ |
| Diastolic BP | 0.01 | 0.99 |
| Total cholesterol | 0.414** | 3.8×10^-10^ |
| Triglyceride | 0.205** | 3.8×10^-10^ |
| HDL- cholesterol | -0.400** | 5.7×10^-20^ |
| LDL- cholesterol | 0.534** | 2.6×10^-25^ |
| ApoA1 | -0.491** | 6.5×10^-28^ |
| ApoB | 0.746** | 3.3×10^-26^ |
| Current smoking | -0.038 | 0.08 |
| Alcohol intake | -0.021 | 0.344 |
| Hypertension | 0.026 | 0,242 |
| Diabetes | 0.033 | 0.139 |
| P-value by pearson correlation analysis**. Correlation is significant at the 0.01 level (2-tailed). | | |

|  |  | | | |  |
| --- | --- | --- | --- | --- | --- |
| Table 2. Multiple linear regression analysis of the apoB/A1 ratios as for important covariates. | | | | | |
| Variable | | Standardized Coefficients | Standard Error | *P*-value* | |
| Age | | 0.001 | 0.001 | 0.701 | |
| Sex | | 0.021 | 0.017 | 0.305 | |
| BMI | | 0.006 | 0.002 | 0.68 | |
| Systolic BP | | 0.001 | 0.005 | 0.231 | |
| Total cholesterol | | 0.192 | 0.01 | 4.32×10^-9^ | |
| Triglyceride | | 0.075 | 0.005 | 2.63×10^-4^ | |
| HDL- cholesterol | | -0.122 | 0.027 | 2.17×10^-7^ | |
| LDL- cholesterol | | 0.365 | 0.012 | 4.65×10^-33^ | |
| Current smoking | | -0.014 | 0.003 | 0.312 | |
| *P-value by multiple linear regression analysis | | | | | |

**Figure 1. Histogram distribution of ApoB/A1 ratio in our population.**

**Figure 2.** **Association between ApoB/A1 ratio and** **total cholesterol/HDL and Framingham Risk Score.** Linear regression, including a quadratic term, of total cholesterol/HDL (A) and Framingham Risk Score (B) on ApoB/A1 ratio. The regression line and the coefficient of determination (R^2^) with adjustment for age, sex, BMI, smoking, drinking, hypertension, and diabetes, are also depicted.

**Figure 3. Associations Between Tertiles of ApoB/A1 ratio and LDL and TG levels.** (A) Tertiles of ApoB/A1 ratio and plasma LDL levels. Lowest tertile (n = 741), middle tertile (n = 761), highest tertile (n = 760). (B) Tertiles of ApoB/A1 ratio and plasma TG levels. TG = triglyceride. LDL= low-density lipoprotein cholesterol.

**Figure 4. Adjusted odds ratios [95% confidence interval (CI)] for coronary artery disease patients with the diagnosis determined by angiography according to quartiles of ApoB/A1 ratios in subgroups.** ^#^ P for interaction: ApoB/A1 ratios quartiles as continuous variables and added these variables to the aforementioned multivariate model. The OR values were adjusted with sex, age, BMI, smoking, drinking, hypertension and diabetes.

**Figure 5. Associations between tertiles of ApoB/A1 ratio and CHD diseas severity.** A, The association between tertiles of apoB/A1 ratio and Gensini score. Analysis was adjusted for age, sex, somoking, drinking, hypertension, diabetes, and body mass index. Lowest tertile (n = 741), middle tertile (n = 761), highest tertile (n = 760). B, Number of cardiovascular and cerebrovascular disease manifestations and ApoB/A1 ratio in CHD patients. The boxes represent median ApoB/A1 ratio with 25th and 75th percentiles. The lower and upper whiskers represent 10th and 90th percentiles, respectively. Median ApoB/A1 ratio of CHD patients is gradually increased according to the number of atherosclerotic disease manifestations: none, 1 (Previous myocardial infarction, Heart failure, History of cerebrovascular disease), 2 (any combination of the previous), or 3. NS, no significant.

|  |
| --- |

**Figure 1.**


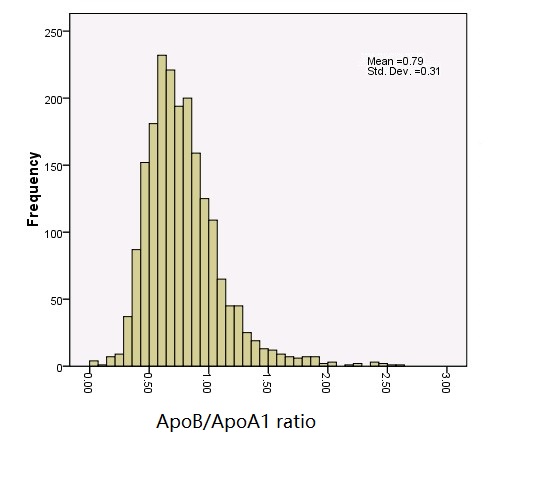


**Figure 2.**


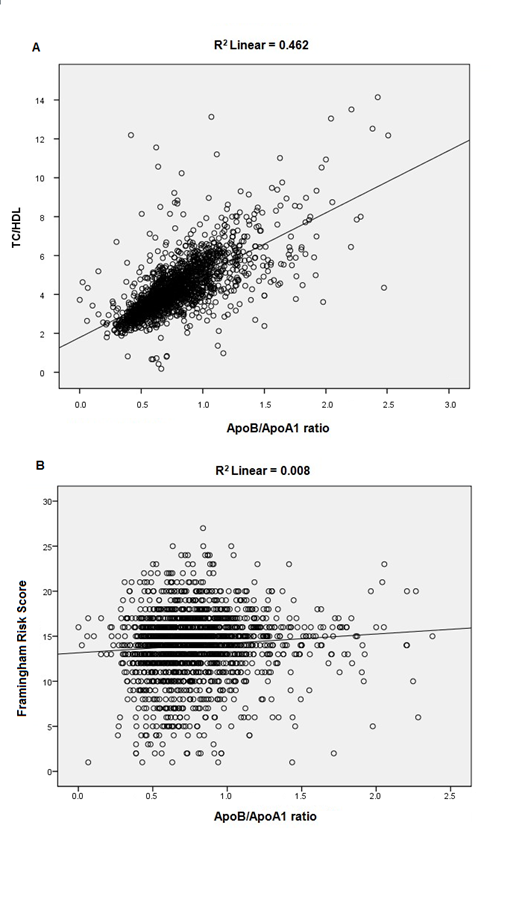


**Figure 3.**


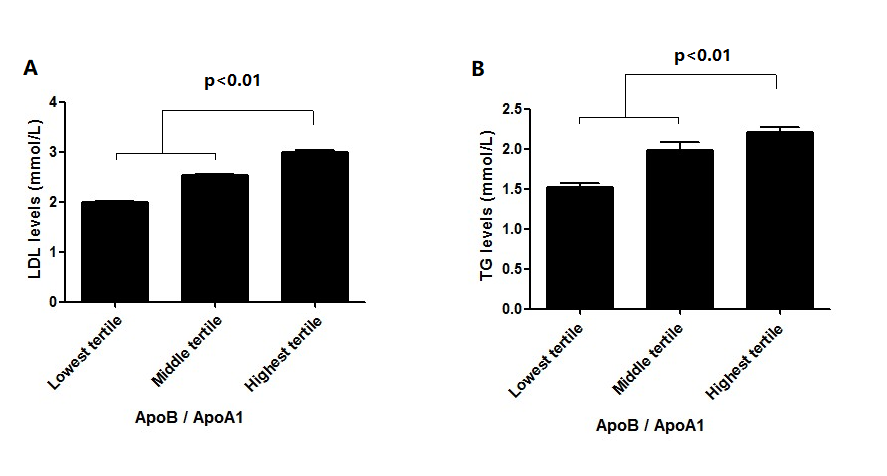


**Figure 4.**


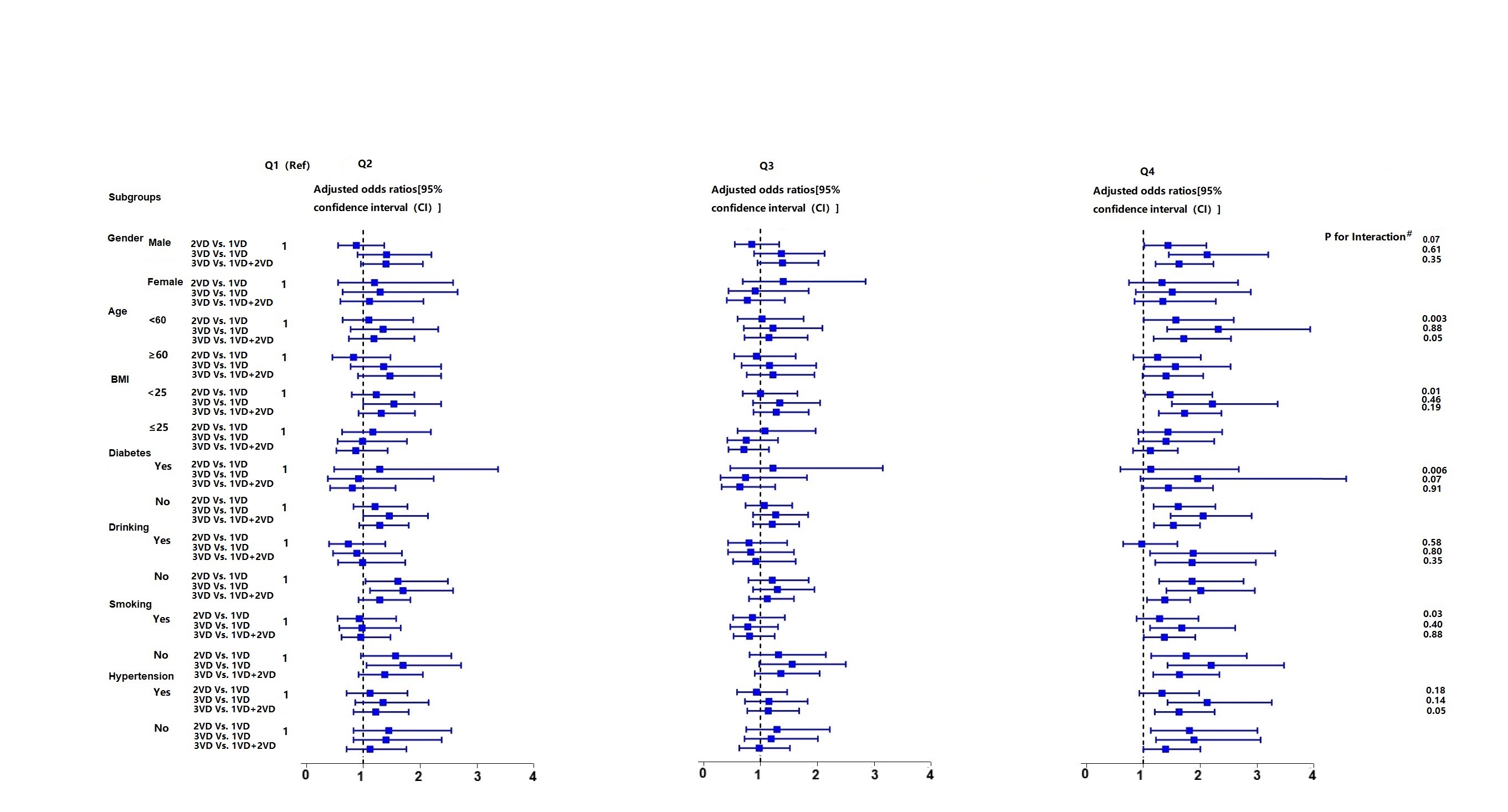


**Figure 5.**


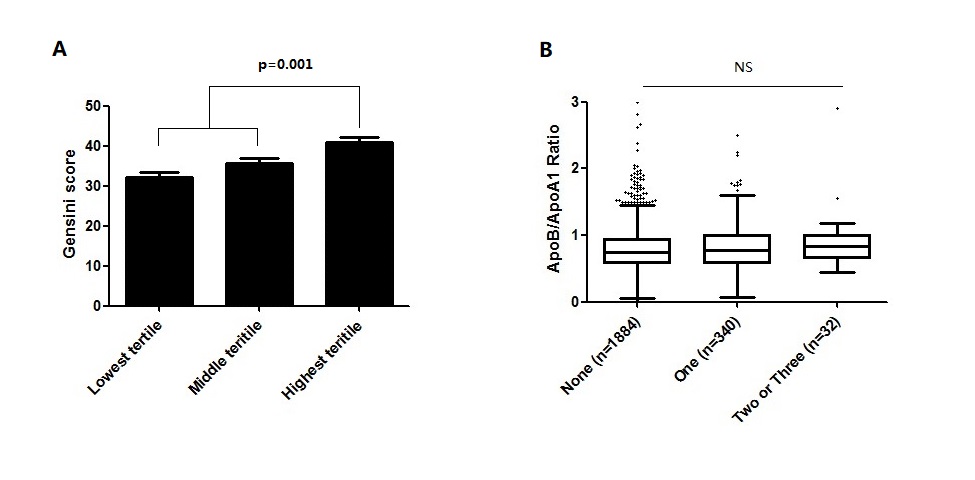

Supplement: Supplementary file 1 — Additional file 1. Table S1. ApoB/A1 ratio with demographic characteristics and cardiovascular risk factors within CHD patients. Table S2. Multiple linear regression analysis of the apoB/A1 ratios as for important covariates. Figure S1. Histogram distribution of ApoB/A1 ratio in our population. Figure S2. Association between ApoB/A1 ratio and total cholesterol/HDL and Framingham Risk Score. Figure S3. Associations Between Tertiles of ApoB/A1 ratio and LDL and TG levels. Figure S4. Adjusted odds ratios [95% confidence interval (CI)] for coronary artery disease patients with the diagnosis determined by angiography according to quartiles of ApoB/A1 ratios in subgroups. Figure S5. Associations between tertiles of ApoB/A1 ratio and CHD diseas severity. [file 12944_2019_1144_MOESM1_ESM.doc]
